# Enterovirus-Human-Rhinovirus Infection Leading to Acute Respiratory Distress Syndrome: A Case Report

**DOI:** 10.7759/cureus.31615

**Published:** 2022-11-17

**Authors:** Arthur Cecchini, Ahmad Othman, Kirandeep Kaur, Austin Richardson, Amanda Cecchini

**Affiliations:** 1 Internal Medicine, East Tennessee State University Quillen College of Medicine, Johnson City, USA; 2 Internal Medicine, St. Bernards Healthcare, Jonesboro, USA; 3 Pulmonary and Critical Care Medicine, Eastern Virginia Medical School, Norfolk, USA

**Keywords:** acute respiratory distress syndrome (ards), acute respiratory distress syndrome, high-flow nasal cannula, high-flow nasal cannula (hfnc), dexamethasone, acute hypoxemic respiratory failure, ground-glass opacities, secondary pneumonia, sars-cov-2, rhinovirus

## Abstract

Enterovirus-human-rhinovirus (EV-HRV) is best known to cause the “common cold” and asthma exacerbations. Simple bronchitis and community-acquired pneumonia related to EV-HRV are also well documented. Scattered reports of rhinovirus causing acute respiratory distress syndrome (ARDS) have been published, yet the causality between recent SARS-CoV-2 pneumonia and severe ARDS secondary to EV-HRV has not been well defined. This case presents a 67-year-old male who was unvaccinated against SARS-CoV-2 with a past medical history of chronic obstructive pulmonary disease, who recently experienced a mild-to-moderate case of SARS-CoV-2 pneumonia, which was treated with dexamethasone and remdesivir. He was discharged to an inpatient psychiatric facility on as-needed oxygen via nasal cannula. Three weeks later, he experienced an episode of presyncope and was readmitted to the hospital. He then began to require increasing levels of supplemental oxygen via a high-flow nasal cannula. A real-time polymerase chain reaction respiratory pathogen panel was positive for EV-HRV. Computed tomography of the chest revealed extensive ground-glass opacities. Further workup for bacterial and fungal pneumonia was negative. Repeat SARS-CoV-2 testing was also negative. He required several days of supplemental oxygen via a high-flow nasal cannula. He received a short course of broad-spectrum antibiotics and a 10-day course of high-dose dexamethasone. Ultimately, he fully recovered, did not require further supplemental oxygen, and was discharged on room air.

## Introduction

In recent years, other respiratory viral pathogens have been overshadowed by coronavirus disease 2019 (COVID-19), although these organisms still play an important role in human disease. Enterovirus-human-rhinovirus (EV-HRV) is best known for its ability to cause upper respiratory tract illness and simple bronchitis [1]. Asthma and chronic obstructive pulmonary disease (COPD) exacerbations are also well documented [1,2].

Several cases have been published linking EV-HRV to acute respiratory distress syndrome (ARDS) [3-6], though we could not locate a case of severe EV-HRV ARDS occurring after recovery from a nonsevere case of SARS-CoV-2 pneumonia.

The clinical picture of EV-HRV infection may range from asymptomatic infection, cough and nasal congestion [1], wheezing and dyspnea [2,7,8], to hospitalization requiring supplemental oxygen [1,9], or much less commonly lead to fulminant respiratory failure requiring mechanical ventilation [3-6].

Diagnosis of EV-HRV is most often with real-time polymerase chain reaction (RT-PCR) collected via a nasopharyngeal swab [9]. As with other causes of ARDS, diffuse ground-glass opacities are often seen on computed tomography (CT) [10,11].

There is no specific treatment for EV-HRV-related ARDS, though dexamethasone has gained traction recently for the general treatment of ARDS [10-12]. General ARDS protocol should be followed in those requiring mechanical ventilation [10,13].

## Case presentation

**Figure 1:**
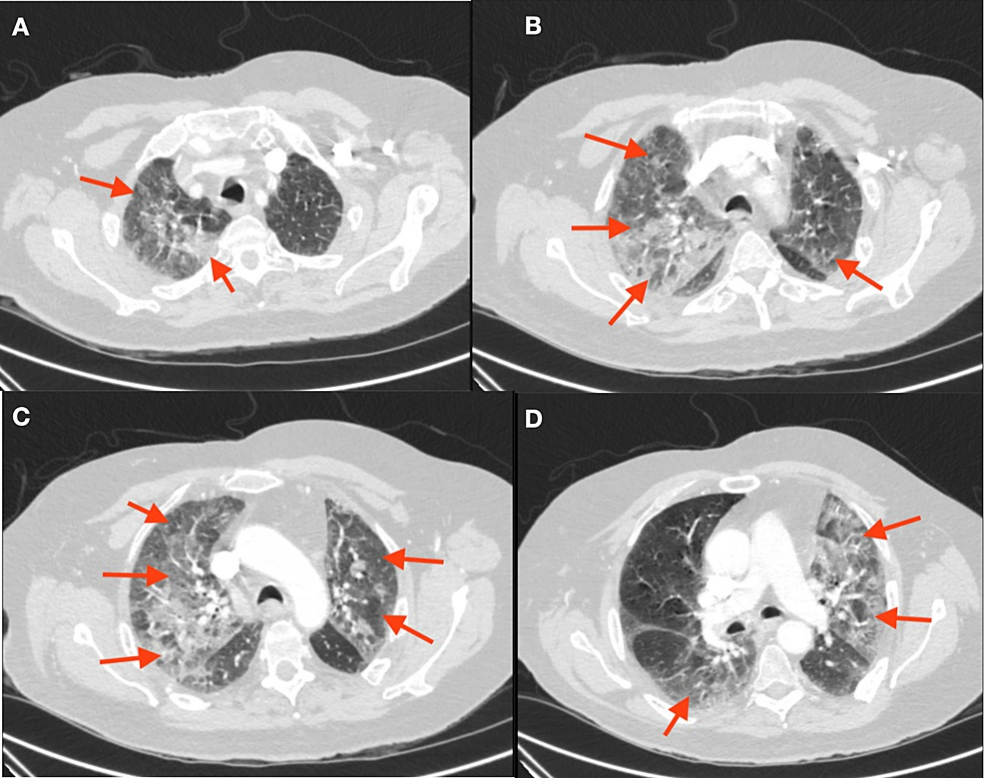
Contrast-enhanced CT of the chest showing diffuse multifocal airspace disease bilaterally

A 67-year-old male with a past medical history of COPD, remote pulmonary embolism on rivaroxaban, well-controlled diabetes mellitus type 2, paranoid schizophrenia, generalized anxiety disorder, and hypertension presented to the hospital for near syncope. Three weeks prior, he was hospitalized for COVID-19 and was given dexamethasone, remdesivir, and supplemental oxygen. His oxygen requirements peaked at 4 liters per minute (LPM), and he was discharged on 2 LPM. During the stay, he had a contrast-enhanced CT of the chest performed showing multi-focal airspace disease bilaterally, chronic elevation of the left hemidiaphragm, and mild dilatation of the main pulmonary artery (Figure 1). He was discharged on dexamethasone to complete a 10-day course.

On this admission, the patient had only fatigue and lightheadedness. He denied any chest pain or productive cough. The patient's vital signs and physical examination were unremarkable on admission.

Laboratory studies, including B-type natriuretic peptide (BNP), complete blood count (CBC), and complete metabolic panel (CMP) were unremarkable on admission. He received 1 liter of lactated Ringer's solution in the emergency department due to concern for volume depletion.

**Figure 2:**
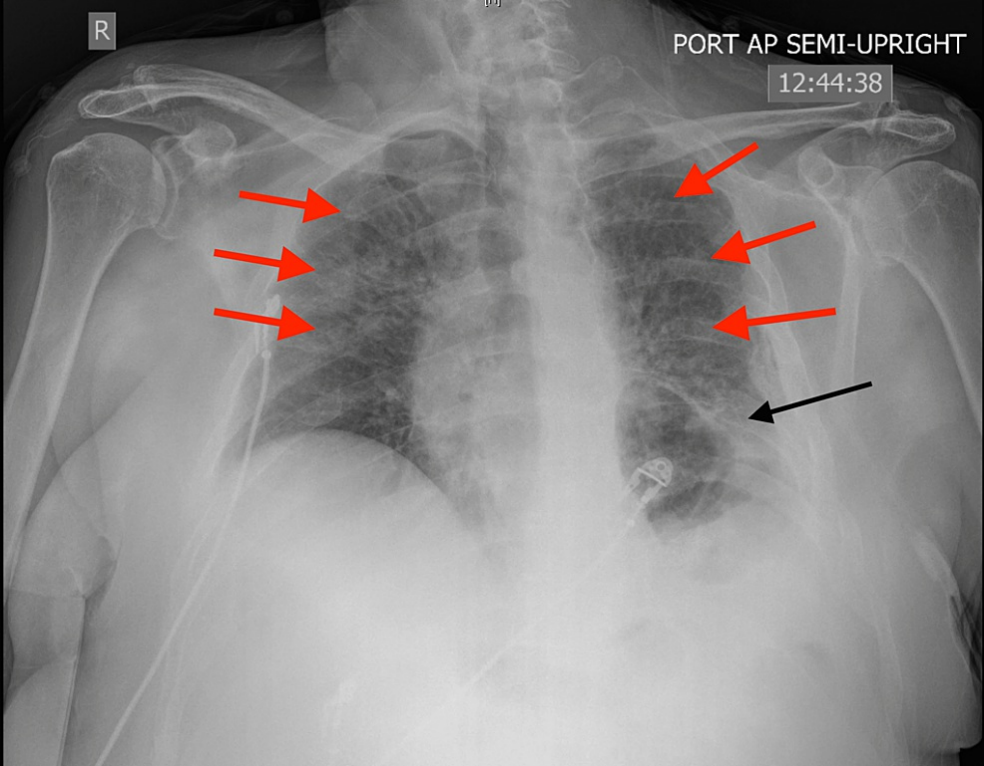
Anteroposterior chest radiograph showing chronic interstitial markings (red arrows), chronically elevated left hemidiaphragm (black arrow), but no acute process

One view chest radiography performed on admission showed chronic interstitial markings, and a chronically elevated left hemidiaphragm, but no acute process (Figure 2).

Orthostatic vital signs showed blood pressures of 123/53 mmHg supine, 118/59 mmHg seated, and 106/64 mmHg standing. The patient’s atenolol was subsequently discontinued and his olanzapine, which had recently been increased to 25 mg, was decreased to 10 mg. The patient's symptom of lightheadedness was most likely related to recent dosage changes in his medications. He symptomatically improved after these adjustments were made.

Four days into admission, the patient’s oxygen requirements began to increase rapidly. An arterial blood gas (ABG) analysis was performed on 8 LPM per nasal cannula (Table 1).

**Table 1:** Arterial blood gas analysis results PCO2: partial pressure of carbon dioxide; PO2: partial pressure of oxygen; HCO3: bicarbonate.

Arterial blood gas analysis	Patient values	Reference values
pH	7.43	7.35-7.45
PCO2 (mmHg)	41.4	35-45
PO2 (mmHg)	54	82-92
HCO3 (mmol/L)	26.7	22-26

A repeat physical exam showed a patient in moderate respiratory distress, on 8 liters of oxygen per minute, with increased work of breathing, and scattered rhonchi. There was no jugular venous distention, S3 heart sound, or pitting edema.

**Figure 3:**
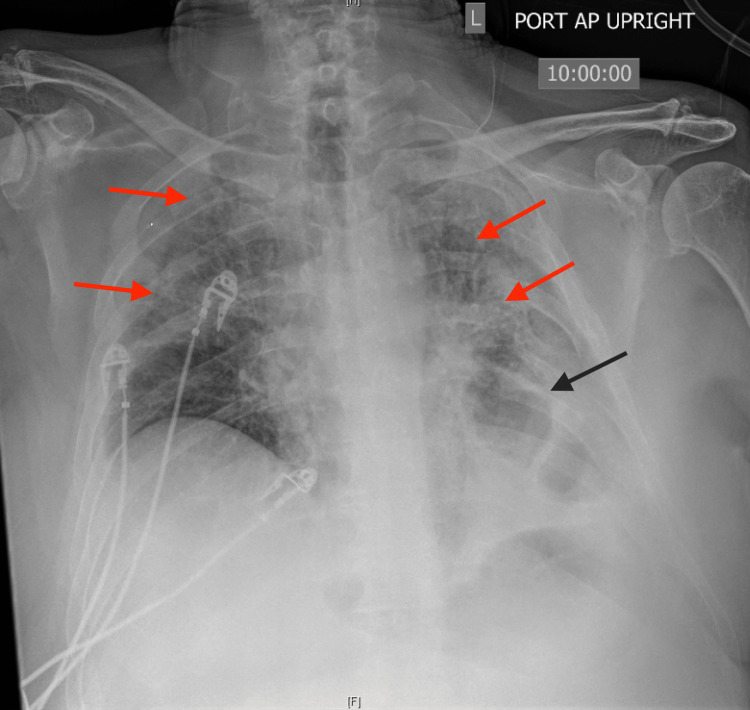
Repeat anteroposterior chest radiography performed four days into admission showing decreased lung volumes, chronically elevated left hemidiaphragm (black arrow), and bilateral airspace opacities (red arrows) suspicious of bilateral multifocal pneumonia

A repeat one-view chest radiograph was performed showing decreased lung volumes, bilateral airspace opacities suspicious of bilateral multifocal pneumonia, and a chronically elevated left hemidiaphragm (Figure 3).

The patient was started on empiric antimicrobial therapy with vancomycin and cefepime due to concern for secondary bacterial pneumonia. The respiratory viral panel returned positive for rhinovirus/enterovirus. Methicillin-resistant *Staphylococcus aureus* (MRSA) nasal screening also returned positive. The procalcitonin and white blood count returned elevated. The rest of the repeat CBC, CMP, and BNP were unremarkable. An extensive evaluation for pneumonia was performed and otherwise returned negative (Table 2).

**Table 2:** Laboratory evaluation

Laboratory studies	Patient values	Reference values
White blood cell count (10^9^/L)	14.4	3.5-10.5
Procalcitonin (ng/mL)	4.40 (8.20 on the previous admission)	<0.05 ng/mL: low risk of sepsis; 0.5-2.0 ng/mL: clinical correlation required; >2.0 ng/mL: high risk of sepsis
Methicillin-resistant *Staphylococcus aureus* (MRSA) nasal polymerase chain reaction (PCR)	Positive	Negative
Respiratory virus panel PCR	Positive for human rhinovirus/enterovirus; negative for respiratory syncytial virus, influenza A/B, SARS-CoV-2, and multiple other respiratory pathogens	Negative
Human immunodeficiency virus 1/2 antibody	Negative	Negative
1,3-beta-D-glucan (pg/mL)	Negative	Negative
Aspergillus antigen enzyme immunoassay (EIA)	Negative	Negative
Cryptococcal antigen	Negative	Negative
Blastomyces antibody, serum	Negative	Negative
Histoplasma antigen, urine	Negative	Negative
Coccidiosis antibody	Negative	Negative
Sputum culture and sensitivity	Normal respiratory flora only	Negative
Streptococcus pneumoniae antigen, urine	Negative	Negative
Legionella antigen, urine	Negative	Negative

**Figure 4:**
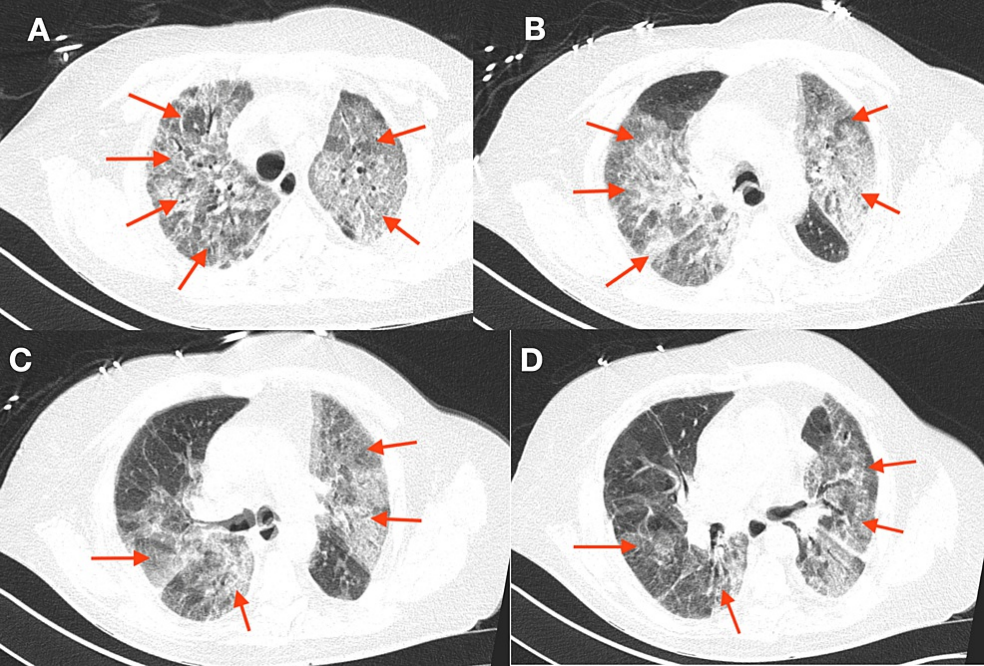
CT of the chest showing new extensive ground-glass opacities

A noncontrast CT of the chest was performed showing extensive ground-glass opacities in the lungs (Figure 4).

A pulmonology consultation was requested for the consideration of bronchoscopy. The procedure was ultimately deferred, as this would have required elective intubation, and suspicion of ARDS secondary to EV-HRV was high. His peak oxygen requirement during admission was on a high-flow nasal cannula (HFNC) at 40 LPM and a fraction of inspired oxygen (FIO2) of 1.0. The antimicrobial therapy was discontinued as there was no improvement and the sputum culture returned only with normal flora. The patient was placed on dexamethasone at 20 mg daily for five days, followed by 10 mg for five days per the hospital’s acute respiratory distress protocol. He was also provided albuterol with ipratropium as needed for wheezing. Rapid improvement was noted once dexamethasone was initiated, his oxygen requirements subsequently declined to room air, and he was able to make a full recovery.

## Discussion

EV-HRVs are positive-sense single-stranded RNA viruses. Previously, this group was considered to be “common cold” viruses, yet recent human volunteer studies have shown that they may cause severe infections, especially in patients with underlying lung disease [1]. EV-HRVs are known to cause exacerbations of reactive airway disease in both children and adults [2], though more recently, they have been associated with ARDS [3-6].

The clinical presentation of EV-HRV varies with many patients remaining asymptomatic or having mild upper respiratory tract symptoms such as nasal congestion, cough, and fatigue [1]. Wheezing and dyspnea may occur in patients with reactive airway disease, ranging from mild symptom exacerbation to near-fatal asthma exacerbations [2]. Patients with COPD may also experience wheezing and dyspnea, ranging from mild to severe [7,8]. EV-HRVs are also often detected on laboratory testing in patients presenting with fever, productive cough, and dyspnea, subsequently diagnosed with community-acquired pneumonia [9].

The most common diagnostic technique for EV-HRV is RT-PCR. As RT-PCR cannot differentiate rhinovirus from enterovirus, a positive result is often reported as rhinovirus/enterovirus [1]. Often, EV-HRV is detected incidentally when an evaluation of pneumonia is performed [9].

In patients with EV-HRV-associated ARDS, progressive dyspnea and hypoxemia are present [3-6]. Imaging most often reveals the typical findings of ARDS with diffuse bilateral infiltrates on plain radiography [3,4,10], and heterogeneously extensive infiltrates and ground-glass opacities on CT [11]. CT also assists with excluding other causes of hypoxemia, including pulmonary embolism, masses, atelectasis, and pleural effusions [10].

Treatment for EV-HRV-related ARDS is like that of other causes with correction of hypoxemia, permissive hypercapnia, prone positioning, and more recently, the use of glucocorticoids such as dexamethasone [5,10-12]. Low tidal volume ventilation at 4-6 ml/kg of ideal body weight is recommended for those requiring mechanical ventilation [10,13]. No specific antiviral therapy is currently available for EV-HRV [1].

We believe this patient's respiratory failure was due to ARDS caused by EV-HRV, as the extensive workup performed was otherwise unrevealing. The MRSA polymerase chain reaction (PCR) was likely showing only chronic carriage as the patient's sputum gram stain and culture did not reveal the growth of MRSA. The ground-glass patterns noted on CT imaging also seemed to represent viral pneumonia. Rapid improvement was also noted after the initiation of dexamethasone, which further supports this diagnosis.

We found a few documented reports of EV-HRV causing ARDS [3-6] but were unable to locate a case occurring shortly after a patient recovered from a comparatively mild SARS-CoV-2 infection. The mildness of this patient's SARS-CoV-2 infection when contrasted to his EV-HRV infection makes this case unusual.

## Conclusions

We believe this case highlights that EV-HRV is another potentially dangerous pathogen that should not be overlooked as a potential etiology behind ARDS. In addition, SARS-CoV-2 infection may allow for severe secondary viral pneumonia and ARDS to develop after patients have recovered from the initial illness. The use of the respiratory viral panel PCR should be strongly considered when the etiology behind ARDS is unclear, and future studies detailing the clinical utility of this diagnostic test would be beneficial. This case is unusual as our patient experienced a relatively mild COVID-19 infection, even though he was unvaccinated, followed by a severe case of ARDS related to EV-HRV.
